# Contemporary Management of the Aortic Valve—Narrative Review of an Evolving Landscape

**DOI:** 10.3390/jcm14010134

**Published:** 2024-12-29

**Authors:** Srihari K. Lella, Brandon E. Ferrell, Tadahisa Sugiura

**Affiliations:** 1 Division of Cardiothoracic Surgery, Department of Cardiothoracic and Vascular Surgery, Montefiore Medical Center, Bronx, NY 10467, USA; slella@montefiore.org (S.K.L.); bferrell@montefiore.org (B.E.F.); 2 Montefiore Medical Center, Department of Cardiothoracic and Vascular Surgery, Medical Arts Pavilion, 3400 Bainbridge Road, 5th Floor, Bronx, NY 10467, USA

**Keywords:** aortic stenosis, minimally invasive aortic valve replacement, transcatheter aortic valve replacement, aortic valve models

## Abstract

**Background:** Aortic valve replacement has undergone novel changes in recent decades, providing not only a multitude of procedural options but expanding the treatable patient population. Specifically, a number of minimally invasive and interventional treatment options have allowed for the treatment of high and prohibitive risk surgical patients. Further, technology is allowing for the development of innovative surgical and transcatheter valve models, which will advance the treatment of aortic valve disease in the future. **Objective:** Here, we choose to describe the modern aortic valve replacement techniques and the available valves and designs.

## 1. Introduction

Aortic valve disease includes two primary pathologies: aortic stenosis (AS), causing narrowing of the valve orifice that limits antegrade flow, and aortic regurgitation (AR), resulting in retrograde flow through the valve. The result of these pathologies in the long-term can cause myocardial remodeling that can affect cardiac output, increase wall stress, cause arrhythmias, and result in ischemia [1]. Moderate and more severe AR has a prevalence of 0.5% in the US population, with increasing prevalence with advanced age and the presence of a bicuspid aortic valve [2,3]. Similarly, AS is relatively uncommon at younger ages in developed countries for patients without congenital abnormalities. Severe AS has a prevalence of 3–4% after age 70 and close to 10% after age 80 [4,5]. The disease burden of these pathologies is only expected to increase in the coming years as the elderly population continues to increase.

Surgical aortic valve replacement (SAVR) has been the standard treatment option for patients with aortic valve disease. Their history dates back to the 1950s when the first mechanical heart valve was developed, the ball-in-cage valve [6]. During ventricular contraction, the higher pressure allows the ball to move up into the wider portion of the cage/aorta, allowing for blood to move around the ball [6]. The first mechanical aortic valve replacement occurred in 1961 [6]. However, ball-in-cage valves were eventually replaced with tilting disc valves and subsequently into the bileaflet valves, which are the predominantly utilized valves today [6]. Simultaneously, due to the interest in limiting the need for anticoagulation, tissue valve options were also explored. Initially, donor grafts were implanted from human allografts and from pig/cow xenografts [7]. However, due to the limitation in the number of available human allografts, as well as the issue of durability of xenografts, bioprosthetic valves were created from xenograft tissue that have been treated with glutaraldehyde to decrease antigenicity [7]. While durability of the bioprosthetic valves remains an issue from calcification and structural deterioration, there is ongoing investigation to improve these valves from these shortcomings [7].

SAVR has traditionally been performed via a median sternotomy, but in recent years, it is also being conducted in a minimally invasive approach. Advancements in technology have also allowed for the development of a transcatheter aortic valve replacement (TAVR) approach for aortic stenosis. While TAVR was initially utilized for high-risk surgical patients, further expansion of the patient population now allows for the use of this method in moderate and low-risk patients as well. With these numerous approaches for aortic valve replacement and a variety of valve types, a number of valve designs have also been developed. In this review, the modern aortic valve replacement strategies and various valve designs will be discussed.

## 2. Aortic Valve Replacement Techniques

### 2.1. Minimally Invasive Aortic Valve Replacement Techniques

**Figure 1:**
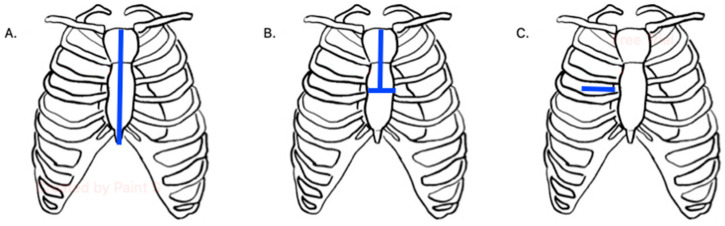
Depiction of various approaches to surgical aortic valve replacement. (**A**) Full median sternotomy, (**B**) Upper hemisternotomy with inverted T-shape, (**C**) Right anterior mini-thoracotomy.

Although aortic valve replacement (AVR) has historically been performed via a median sternotomy, minimally invasive AVR techniques have been developed over the years to reduce morbidity, decrease hospital length of stay and costs, and improve cosmesis [8,9,10]. Approaches to minimally invasive AVR surgical techniques include partial sternotomy, right anterior mini-thoracotomy, and robot-assisted surgery (Figure 1). While these techniques do require a steep learning curve, they can be especially useful to treat patients with significant co-morbidities. As such, there continues to be a paradigm shift, especially in higher volume regions and institutions, to minimally invasive approaches [11].

Several types of partial sternotomy techniques have been described and can be categorized based on the portion of the sternum that is divided. These include upper, middle, and lower hemisternotomies. The most commonly used is the upper hemisternotomy, given its similarity to a full median sternotomy. These typically include an inverted T- and J-shaped mini-sternotomies, which can be made via skin incisions as small as 4–5 cm in length. Lower hemisternotomy is the next most common, which include T-shape and inverted L-shape sternotomies. Finally, middle hemisternotomy is the least commonly utilized with H-shape and reverse C-shape sternotomies. Regardless of the mini-sternotomy approach, central cannulation is feasible, internal mammary arteries are preserved, and the ascending aorta can be well exposed. Several drawbacks to this approach include difficulty with de-airing, limited retrograde cardioplegia administration, and post-operative limitation in upper extremity mobilization [12,13].

Right anterior mini-thoracotomy typically involves a 5–8 cm incision over the second or third intercostal space. It is a technically difficult approach but avoids division of the sternum and ribs. Additionally, it provides preservation of the upper rim allowing for postoperative mobility of the upper limbs. Central cannulation can sometimes be feasible with this approach, but peripheral cannulation is often required for this approach [12,13,14].

Finally, robotic-assisted aortic valve replacement is approached via 4–5 small incisions over the right intercostal spaces. In addition to the robotic port incisions, an additional incision is typically utilized as a working/assistant port. The advantages and disadvantages are similar to those of an anterior mini-thoracotomy, including peripheral cannulation and cosmesis. Additionally, the right internal mammary artery can more often be preserved with this approach. However, the robotic approach requires institutional resources to support a robotic program, and a surgeon experienced with the robot [15,16].

Although studies comparing AVR via full median sternotomy vs. minimally invasive approaches have largely been restricted to registries and single center case series, minimally invasive AVR has been established as a safe and effective alternative. One of the largest benefits of minimally invasive AVR is decreased postoperative bleeding and need for transfusions [17,18,19,20,21]. Dogan et al. demonstrated significantly lower post-operative chest tube output for their minimally invasive AVR patients [17]. Further, a meta-analysis that included 26 studies showed reduced blood loss in the minimally invasive AVR patients within the first 24 h after surgery [18]. While Stamou et al. showed no difference in transfusion requirements between the different surgical approaches, Gilmanov et al. and Bonacchi et al. have demonstrated decreased post-operative transfusion requirements after minimally invasive AVR [19,20,21]. Another benefit of minimally invasive AVR is faster recovery with a shorter hospital stay, thought to be due to the decreased sternal/thoracic trauma [12]. Several meta-analyses have demonstrated a decreased ventilation time, ICU length of stay, and total hospital length of stay for minimally invasive AVR patients [22,23]. Furthermore, Sharony et al. showed that a greater proportion of minimally invasive patients were discharged home than to rehabilitation facilities compared to full median sternotomy patients [24]. An additional advantage of the smaller incision and decreased sternal trauma is the reduced postoperative pain. Yamada et al. and Candaele et al. both demonstrated diminished pain for minimally invasive AVR patients with improved quality of life [25,26]. As would be expected, however, this is dependent on how the tissue is handled and the traction placed on other structures, such as the intercostal space, during the minimally invasive approaches [12]. Finally, wound complications between the different approaches continue to be studied. Sternal wound infections have not been shown to be significantly different between the hemi and full sternotomy patients [18,27]. With certain approaches, central cannulation is not feasible and, typically, femoral cannulation is utilized, which does involve a risk of groin wound complications [28].

### 2.2. Transcatheter Aortic Valve Replacement

TAVR, although also minimally invasive, does not involve surgical replacement of the aortic valve; instead, it allows for the delivery of a prosthetic valve into the aortic valve position from a peripheral location. For the past two decades, there has been a significant rise in TAVR procedures as technology and procedural proficiency have advanced with time. Since 2019, TAVR has been performed more frequently than SAVR in the US [29]. Further, because of the improved technology, valve-in-valve TAVR is increasingly feasible, decreasing the need for redo surgery after previous biological surgical valve replacement [30]. With recent studies demonstrating its non-inferiority over SAVR in even low-risk surgical patients, its use will continue to expand, especially for patients desiring a less invasive approach [31].

**Figure 2:**
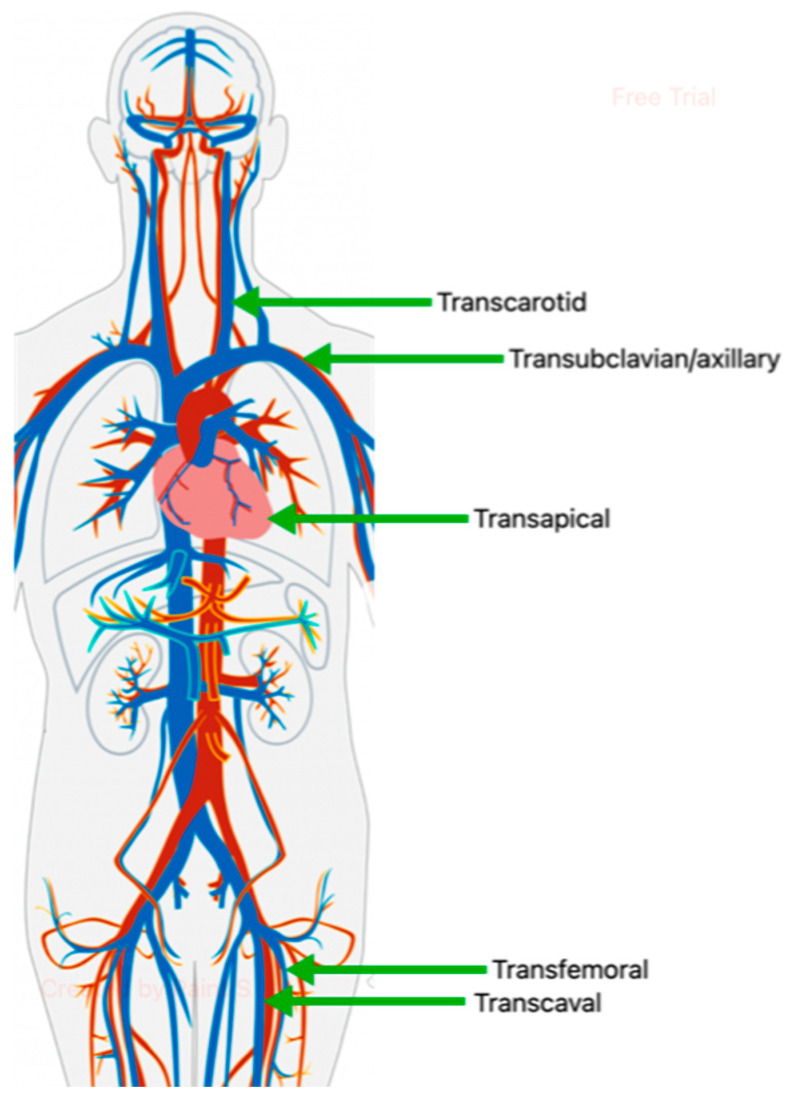
Schematic depicting various access sites for approach to transcatheter aortic valve replacement.

Multiple access points have been described for the delivery of the transcatheter aortic valve, each with its own benefits and drawbacks. Transfemoral is the most common and preferred access site as it also has the advantage of being completely percutaneous [32]. This has the added advantage of potentially minimizing sedation as it is occasionally being performed with local anesthesia [33]. However, this access can be disadvantageous in cases of significant peripheral arterial disease and/or tortuous iliofemoral vessels [34]. Other access points, which are rarely used but are available in special circumstances, include transapical, transaxillary/subclavian, transaortic, transcarotid, and transcaval (Figure 2).

Deciding between SAVR and TAVR requires a tailored approach to the patient based on risk profile, type of valve desired, and need for concomitant procedures. Age is also a vital factor in determining appropriateness between the two procedures. The most recent guidelines by the American College of Cardiology/American Heart Association and the European Society of Cardiology/European Association for Cardio-Thoracic Surgery recommend SAVR for younger patients with acceptable surgical risk profile while TAVR is preferred for older patients and/or patients with higher risk profiles [31,35]. While the specific age cutoffs are different between the two sets of guidelines, there is an intermediate age group, between 65–80 years, where a more nuanced approach to the type of AVR is required [31,35]. This should specifically take the patient’s surgical risk profile, co-morbidities, and life expectancy into account. Additionally, patient preference should also be considered in deciding between SAVR and TAVR. Specifically, the durability of SAVR and TAVR should be extensively discussed with the patient. Long-term data on TAVR valve durability is currently limited as these were initially utilized for high-risk patients with shorter life expectancy [36]. As such, long-term data for TAVR in younger patient populations continues to be established. Mid-term (5-years) outcomes currently demonstrate lower rates of bioprosthetic valve dysfunction in patients who have undergone TAVR compared to SAVR [37,38,39]. Thyregod et al. also recently published data on 10-year outcomes of TAVR vs. SAVR durability in a younger patient group [40]. Patients in the TAVR group had a significantly lower rate of structural valve deterioration [40]. On the other hand, patients with moderate-to-severe paravalvular leaks were significantly higher in the TAVR group [40]. However, bioprosthetic valve failure, which is defined as valve-related death, severe hemodynamic structural valve deterioration, or aortic valve reintervention after diagnosis of bioprosthetic valve dysfunction, was not significantly different between the TAVR and SAVR groups [40]. TAVR patients had a larger effective orifice area and lower transvalvular gradients compared to SAVR patients [40]. As longer-term data in younger patients becomes available, it will help decision-making for this specific patient population. Furthermore, as valve-in-valve and TAVR-in-TAVR technology progresses, treatment options and strategies will continue to evolve and should be considered in determining the optimal strategy for each patient. Table 1 depicts summaries of the pivotal TAVR trials that have been conducted.

When considering TAVR, preoperative planning is essential in choosing the appropriately sized valve and need for any special considerations for vascular access. Multidetector computed tomography (CT) is now considered the gold standard for 3D imaging to determine the aortic annulus size [31,35]. This is necessary to reduce the risk of paravalvular leak while avoiding an oversized valve that has the risk of impinging on conductive tissue [48,49]. The disadvantage to this imaging modality is the need for intravenous contrast, which might be limited in patients with an allergy or kidney impairment. Three-dimensional transesophageal echocardiography has alternatively been shown to correlate well with CT to determine aortic annulus size [50]. Imaging is also essential in determining valve/leaflet calcifications. Some degree of aortic valve calcification is preferred in TAVR as it helps to keep the prosthetic stably housed; however, excessive calcification can negatively impact valve positioning and/or valve expansion, resulting in paravalvular leaks [51,52]. Furthermore, increased calcification may also increase the risk of embolization, which can result in stroke or other peripheral malperfusion, and worsen long-term patient outcomes [51]. Finally, another important consideration for diabetic patients who are planning to undergo TAVR for severe aortic stenosis, a recent study has found that sodium-glucose cotransporter-2 inhibitors were associated with favorable cardiac remodeling with reduced major adverse cardiovascular events at a 2-year follow-up [53].

Important complications resulting from TAVR include vascular access complications, paravalvular leaks, cerebrovascular accidents, conduction abnormalities, and valve failure [54]. Vascular access complications include hematoma, pseudoaneurysm, dissections, occlusion, and perforations. Historically, they have had significantly high incidence due to the larger access sheaths utilized; however, these have generally decreased as sheath sizes have decreased with time, with current rates being below 5% [47,55]. Similarly, paravalvular leaks after TAVR have also had a significant drop in incidence with the advancement in prosthetic design, with modern rates being as low as 2% [56]. The majority of these are mild in severity, but when they are more significant, they have been shown to have worsened mortality rates [57]. Ways to treat paravalvular leaks include balloon dilation of the valve, placement of vascular plugs, repositioning of the valve if improperly positioned, and implantation of another transcatheter valve [58]. Nevertheless, as previously mentioned, careful preoperative assessment of the patient imaging with evaluation of the valve anatomy and calcifications are necessary in mitigating the risk of these leaks. Another significant complication from TAVR is cerebrovascular accidents, which most often occur peri-procedurally [59]. Strokes occurring within 30-days of the procedure have been shown to have up to a six-fold increase in 30-day mortality [60]. While cerebral embolic protection devices are continuing to be studied, they have a variability in effectiveness in reducing the risk of stroke [61,62]. More importantly, antithrombotic therapy, regardless of monotherapy or dual therapy, is essential in reducing stroke rates [31].

Conduction abnormalities is another potential complication from TAVR, particularly from oversized valves due to the compression of the nearby cardiac conduction tissue [48,49]. Compared to women, men have been shown to have a higher need for a permanent pacemaker (PPM) due to greater pre-existing comorbidities as well as the fact that men have larger aortic annulus sizes necessitating larger valve sizes [35,48,49,63]. Additionally, patients with pre-existing conduction abnormalities, specifically right bundle branch block, were more likely to receive PPM after TAVR [49]. The type of prosthetic valve has also shown to affect the risk of conduction abnormalities necessitating PPM. Specifically, self-expanding valves have a higher risk of causing conduction abnormalities compared to balloon-expandable valves [56]. In order to reduce the risk of conduction abnormalities associated with TAVR, it is important to consider the patient’s baseline conduction abnormalities and determine the most appropriate valve type as well as to optimize valve positioning by avoiding deep implantation into the left ventricular outflow tract [64]. Novel methods of valve implantation are actively being studied for both types of valves to determine if the need for post-TAVR PPM can be reduced [65,66]. Finally, as with a surgical implant, TAVR implants can also eventually result in valve failure. Currently, the rate of deterioration at 1 year is 5%, and there is a concern for this to potentially be higher as indications for TAVR continue to be expanded [67]. Nevertheless, it is anticipated that as the device design continues to advance, so will the durability.

### 2.3. New Models of Valves

A number of prosthetic valves exist today, each with variable designs that may make a particular valve more optimal for each patient. Importantly, regardless of the type of valve, there is always a possibility for prosthetic valve disease that can result from hemodynamics, thrombogenicity, and overall durability [68]. Prosthesis-related complications can be minimized by appropriate patient and prosthesis selection with regular post-implant follow-up [68].

Surgical aortic valves include either mechanical or bioprosthetic valves, which have undergone several developmental iterations since the initial ball-in-cage mechanical valve. Modern day mechanical valves include an external valve ring with two central semilunar disks that are attached to the ring via hinges [68]. The disks open up to 90 degrees, allowing for three orifices through which the blood can eject out of the left ventricle in a laminar fashion [68]. As these valves are typically made of titanium and carbon, they require anticoagulation to reduce the risk of thrombogenesis [68,69]. Due to this drawback, biologic valves were developed in conjunction with the mechanical valves. Today, there are stented and stentless versions of these valves that are typically created using porcine, bovine, or equine tissue [69]. Stented valves typically use an outer supporting stent, that is covered with knitted fabric, with either 3 porcine aortic valve leaflets or bovine pericardial leaflets that are mounted onto the stent [68,69]. Stentless prostheses are developed from harvesting whole porcine aortic valves or built using bovine pericardium [68]. While there is significant variability in terms of study designs and valve manufacturers, studies have typically shown comparable valve durability, functional status, and patient mortality between stented and stentless valves [70]. In addition to animal tissue, donor human valves can also be used, but these are less commonly utilized due to limited availability [69]. Regardless of the type of biologic valve, the concern tends to be shorter durability when compared to mechanical valves [68,69,70].

In addition to the above, several valves have either been developed or are currently in development with further specific advantages. With regards to mechanical valves, there is an ongoing need to reduce and eliminate the need for anticoagulation. The TRIFLO heart valve (Novostia, Lausanne, Switzerland) is a mechanical heart valve with a unique tri-leaflet design made of polymer cusps housed in a titanium alloy [71]. It is designed to optimize valve opening and closure, minimizing shear forces [71]. Trials are ongoing in humans, but it is deemed that anticoagulation will not be necessary for these patients [71]. Bioprosthetic valves have also had variable developments in recent years. The Perceval^TM^ valve (Corcym, London, UK) is the first true sutureless valve on the market [72]. The valve is created from bovine pericardium that partially covers a nitinol stent frame, which extends to the sinotubular junction [72,73]. The stent frames have a coating that allows for improved biocompatibility [72,73]. The stent bows into the sinuses of Valsalva and are designed to mimic the movement of the aorta by reducing the stress on the leaflets [72,73]. The sutureless nature allows for shorter cross-clamp times, which can be beneficial for higher risk patients [73]. The Intuity Elite valve (Edwards, Los Angeles, CA, USA) has some similar advantages to the Perceval. It has bovine pericardial leaflets, which have undergone a ThermaFix process to reduce calcification and are housed onto a stainless-steel stent frame [73,74]. The frame extends in a sub-annular direction and is covered with a textured cloth that improves sealing [73,74]. The valve is positioned and deployed via a balloon system and requires three guiding sutures [73,74]. Similar to the Perceval, the Intuity Elite valve reduces cross-clamp time and is feasible through minimally invasive approaches [73,74]. Finally, the Inspiris Resilia (Edwards, Los Angeles, CA, USA) has been developed to also address the issue of durability with bioprosthetic valves [75]. It is created from bovine pericardial tissue that has undergone a phospholipid removal process which, in turn, blocks aldehyde groups known to bind calcium [75]. Furthermore, it has VFit technology that allows for the expansion of an annular stent in a controlled fashion [75,76]. This allows for future valve-in-valve TAVR to be performed in a predictable manner without needing to fracture the bioprosthetic valve [75,76].

TAVR technology is an ever-expanding field with the continued push towards minimally invasive procedures. There are currently three valves approved by the US Food and Drug Administration (FDA): Edwards SAPIEN 3 Ultra (Edwards, Los Angeles, CA, USA), Medtronic EVOLUT FX (Medtronic, Minneapolis, MN, USA), and Abbott Navitor (Abbott, Chicago, IL, USA) [77]. The SAPIEN 3 Ultra is a balloon-expandable trileaflet bovine pericardial valve that is mounted on a cobalt–chromium spring alloy. Additionally, it has an outer seal cuff to reduce the risk of a paravalvular leak [77]. It is an intra-annular valve, which may result in higher residual gradients, that has a low stent profile allowing for uncomplicated access of coronary vessels after TAVR [77]. The EVOLUT FX is a self-expanding valve that is designed from porcine pericardium sutured onto a nitinol frame [77,78]. A sealing skirt is also made with the same porcine pericardium to reduce the risk of a paravalvular leak [77,78]. It is a supra-annular design, which allows for lower gradients due to the increased effective orifice area [77]. In addition, unlike the SAPIEN 3, it is retrievable and repositionable [77]. However, the large frame height with smaller diamond frame lattice makes coronary access more difficult [77]. Of note, the latest version of EVOLUT FX is the EVOLUT FX+, for which data is limited, but it is designed to have a larger frame lattice that allows for improved coronary access during TAVR [79]. Finally, the Navitor is also a self-expanding valve but with leaflets made from bovine pericardium [77,80]. These are sutured onto a titanium-nickel alloy frame that has a similar height as the EVOLUT FX, but a larger cell design, making coronary access more feasible [77,80]. It is similarly retrievable and repositionable, but it does sit in an intra-annular position [77,80]. Importantly, there are no large-scale direct comparisons of the various TAVR valves. Each device has unique features that make them more amenable to certain patient characteristics and anatomy. Understanding these variable features is more essential in order to select and utilize the most appropriate valve for a particular patient.

While the above TAVR valves are all designed for aortic stenosis, transcatheter aortic valve replacement for aortic valve regurgitation is still being established. Currently, the JenaValve (JenaValve Technology, Irvine, CA, USA) and J-Valve (JC Medical Inc., Burlingame, CA, USA) are undergoing studies for FDA approval in the US for aortic valve regurgitation. JenaValve is a porcine pericardial valve mounted onto a nitinol stent that clips onto the native aortic valve leaflets [77,81,82]. It is a self-expanding stent that requires the valve leaflets to be free of calcium to allow for the valve to anchor onto the leaflets [77,81,82]. The J-valve is made utilizing bovine pericardium leaflets that are mounted onto a nitinol stent [77,83]. It also has anchors that allow it to mate with the native aortic valve leaflets [77,83]. Uniquely, it is deployed in a retrograde fashion towards the valve leaflets, which allows for the position to be adjustable prior to complete deployment [77,83].

## 3. Conclusions

Aortic valve replacement is a continually expanding field that has had a number of changes over recent decades. With minimally invasive surgery and transcatheter aortic valve replacement, the treatment options for aortic valve disease has become quite variable. As such, it is important to be aware of the advantages and disadvantages of both the different types of replacement techniques as well as the different valve options when discussing options with the patient.

## 4. Limitations

This is a narrative review on the current trends of aortic valve replacement. This was not intended to be a systematic review and, as such, PRISMA guidelines were not instituted. As such, there could be some unintended bias.

## Figures and Tables

**Table 1:** Transcatheter aortic valve replacement randomized controlled trials.

Trial	Patient Population	Primary Outcomes	Notable Findings
PARTNER 1A [41]	Severe, symptomatic aortic stenosis in high-risk surgical candidates. 699 patients randomized 1:1 to either TAVR with Edwards SAPIEN vs. SAVR in a non-inferiority design.	All-cause mortality.	TAVR was non-inferior to SAVR in terms of all-cause mortality at 1 year. TAVR had higher stroke, paravalvular leaks, and major vascular complications compared to SAVR. SAVR had a higher rate of major bleeding.
PARTNER 1B [42]	Severe, symptomatic aortic stenosis in poor/inoperable surgical candidates. 358 patients randomized 1:1 to either TAVR with Edwards SAPIEN vs. medical therapy (includes balloon aortic valvuloplasty), in a superiority design.	Co-primary endpoints: All-cause mortality and composite endpoint of all-cause mortality or repeat hospitalization for valve or procedure-related deterioration.	TAVR had lower rate of all-cause mortality at 1 year as well as lower rates of all-cause mortality or repeat hospitalization. TAVR had an increased risk of stroke, major bleeding, and major vascular complications compared to medical therapy.
PARTNER 2A [43]	Severe, symptomatic aortic stenosis in intermediate-risk surgical candidates. 2032 patients randomized 1:1 to either TAVR with 2nd generation Edwards SAPIEN XT vs. SAVR in a non-inferiority design.	Composite of all-cause mortality or disabling stroke.	TAVR was non-inferior to SAVR in terms of all-cause mortality and disabling stroke at 2 years. TAVR had an increased risk of major vascular complications, permanent pacemaker implantation, and rates of paravalvular leaks compared to SAVR.
PARTNER 3 [44]	Severe, symptomatic aortic stenosis in low-risk surgical candidates. 1000 patients randomized 1:1 to either TAVR with Edwards SAPIEN 3 vs. SAVR in a non-inferiority and superiority design.	Composite of all-cause mortality, stroke, or rehospitalization.	TAVR had lower all-cause mortality, stroke, and rehospitalization at 1 compared to SAVR. No significant between-group differences of moderate to severe paravalvular leaks, need for permanent pacemaker, or major vascular complications.
NOTION [45]	Severe, symptomatic aortic stenosis in low-risk surgical candidates. 280 patients randomized 1:1 to either TAVR with Medtronic CoreValve vs. SAVR in a superiority design.	Composite of all-cause mortality, stroke, or myocardial infarction.	TAVR was non-inferior SAVR in all-cause mortality, stroke, or myocardial infarction at 1 year. TAVR had an increased need for permanent pacemaker implantation, larger effective orifice area, and increased aortic valve regurgitation compared to SAVR. SAVR had an increased major bleeding risk, cardiogenic shock, acute kidney injury, and new-onset or worsening atrial fibrillation at 30 days compared to TAVR. A limitation of the study is that for the SAVR group, Mitroflow (Sorin Group, Arvada, US) and Trifecta (Abbott, Chicago, US) biological valves were utilized, which are deemed to be outdated with lower durability.
CoreValve US Pivotal High Risk [46]	Severe, symptomatic aortic stenosis in high-risk surgical candidates. 795 patients randomized 1:1 to either TAVR with Medtronic CoreValve vs. SAVR in a non-inferiority design but also powered for a superiority test.	All-cause mortality.	TAVR had lower all-cause mortality at 1 year compared to SAVR. TAVR had a higher rate of major vascular complications, need for permanent pacemaker, and post-procedure paravalvular leak compared to SAVR. SAVR had a higher rate of major bleeding and atrial fibrillation.
SURTAVI [38]	Severe, symptomatic aortic stenosis in intermediate-risk surgical candidates. 1746 patients randomized 1:1 to either TAVR with Medtronic CoreValve or Evolut R vs. SAVR in a non-inferiority design.	Composite of all-cause mortality or disabling stroke.	TAVR was non-inferior to SAVR in all-cause mortality or disabling stroke. TAVR had a higher rate of major bleeding and major vascular complications at 30 days compared to SAVR. SAVR had a higher rate of any stroke at 30 days compared to TAVR.
EVOLUT Low-Risk [47]	Severe, symptomatic aortic stenosis in low-risk surgical candidates. 1468 patients randomized 1:1 to either TAVR with Medtronic CoreValve, Evolut R, or Evolut PRO vs. SAVR in a non-inferiority design.	Composite of all-cause mortality or disabling stroke	TAVR was non-inferior to SAVR in all-cause mortality or disabling stroke at 2 years. TAVR had a lower rate of disabling stroke, major bleeding, atrial fibrillation, and acute kidney injury at 30 days compared to SAVR. TAVR had a higher rate of moderate to severe aortic regurgitation and need for pacemaker implantation compared to SAVR.
